# Distributional anchor regression

**DOI:** 10.1007/s11222-022-10097-z

**Published:** 2022-05-13

**Authors:** Lucas Kook, Beate Sick, Peter Bühlmann

**Affiliations:** 1 Epidemiology, Biostatistics and Prevention Institute, University of Zurich, 8001 Zurich, Switzerland; 2 Institute of Data Analysis and Process Design, Zurich University of Applied Sciences, 8400 Winterthur, Switzerland; 3 Seminar for Statistics ETH Zurich, 8092 Zurich, Switzerland

**Keywords:** Anchor regression, Covariate shift, Diluted causality, Distributional regression, Transformation models, Out-of-distribution generalization

## Abstract

Prediction models often fail if train and test data do not stem from the same distribution. Out-of-distribution (OOD) generalization to unseen, perturbed test data is a desirable but difficult-to-achieve property for prediction models and in general requires strong assumptions on the data generating process (DGP). In a causally inspired perspective on OOD generalization, the test data arise from a specific class of interventions on exogenous random variables of the DGP, called anchors. Anchor regression models, introduced by Rothenhäusler et al. (J R Stat Soc Ser B 83(2):215–246, 2021. 10.1111/rssb.12398), protect against distributional shifts in the test data by employing causal regularization. However, so far anchor regression has only been used with a squared-error loss which is inapplicable to common responses such as censored continuous or ordinal data. Here, we propose a distributional version of anchor regression which generalizes the method to potentially censored responses with at least an ordered sample space. To this end, we combine a flexible class of parametric transformation models for distributional regression with an appropriate causal regularizer under a more general notion of residuals. In an exemplary application and several simulation scenarios we demonstrate the extent to which OOD generalization is possible.

## Introduction

Common methods in supervised statistical learning assume the test data to follow the same distribution as the training data. This is implicitly exploited in, *e.g.,* cross-validation or by randomly splitting a dataset into a training and a test set, which has been demonstrated to be potentially flawed (Efron 2020) due to concept drift or domain shift where new (test) data do not follow the same distribution as the training data. More recently, the problem has been referred to as out-of-distribution (OOD) generalization (Arjovsky et al. 2019). The desire to achieve reliable test predictions under distributional shifts is ubiquitous in many fields of machine learning and statistics, such as transfer learning (Pan and Yang 2009; Rojas-Carulla et al. 2018), domain adaptation (Magliacane et al. 2018), multi-task learning (Caruana 1997), representation learning (Mitrovic et al. 2020) or prediction models in medical statistics (Subbaswamy and Saria 2019). Accordingly, many different formulations of the problem of OOD generalization exist in the literature (a detailed overview can be found in Chen and Bühlmann 2021). We will frame OOD generalization as the problem of robustly predicting an outcome in novel, unseen environments, based on data from a few observed environments and extend on the idea of anchor regression and causal regularization (Rothenhäusler et al. 2021; Bühlmann 2020; Bühlmann and Ćevid 2020) to develop distributional anchor regression. In such a framework, training a model on heterogeneous data is not a disadvantage but rather a necessity.

### Related work

It has been known for decades that a causal model is robust towards arbitrarily strong perturbations on components other than the response (Haavelmo 1943). However, identifying causal structures is not only difficult but employing them for prediction often leads to sub-par prediction performance when the test data contain perturbations of bounded strength (Rothenhäusler et al. 2021). Rothenhäusler et al. introduce linear anchor regression, which allows a trade-off between prediction performance and robustness against shift perturbations of a certain size. The framework of linear anchor regression was extended to deal with nonlinear regression between the response and covariates (Bühlmann 2020). Furthermore, Christiansen et al. (2021) provide a causal framework to decide which assumptions are needed for and to what extent OOD generalization is possible.

**Fig. 1:**
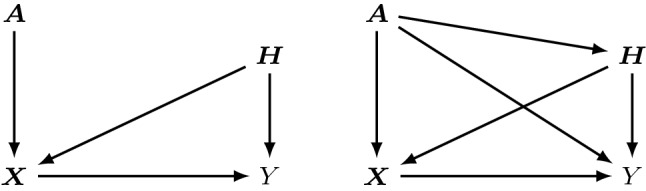
Graphical models for the response variable $Y$, covariates ${\varvec{X}}$ and hidden confounders ${\varvec{H}}$: IV regression with instruments ${\varvec{A}}$ (left) and anchor regression with anchor ${\varvec{A}}$ (right). In anchor regression, ${\varvec{A}}$ is only required to be a source node but is allowed to directly influence response, covariates and hidden confounders

Anchor regression is related to Instrumental Variables (IV) regression. However, the main IV assumption that the instrument ${\varvec{A}}$ does not directly affect some hidden confounding variables ${\varvec{H}}$ is dropped, at the price of non-identifiability of the causal parameter (Angrist et al. 1996). A graphical description of the issue is given in Fig. 1.

### Our contribution

In this work we develop a framework for distributional anchor regression in the broad class of transformation models (TMs, Hothorn et al. 2014). The resulting class of anchor TMs generalizes (non-) linear anchor regression to potentially censored responses and characterizes the full conditional distribution of $Y\vert {\varvec{X}}= {\varvec{x}}$ instead of estimating solely the conditional mean function. While the linear $L_2$ anchor loss can be decomposed into a squared error and causal regularization term penalizing correlation between anchors and residuals, we propose a distributional anchor loss based on the negative log-likelihood and replacing the least-squares residuals by the more general score residuals. The proposed causal regularizer induces uncorrelatedness between the anchors and these score residuals. The resulting procedure is tailored towards protecting against distributional shifts induced by the anchors and naturally interpolates between the unpenalized maximum-likelihood estimate and a solution for which anchors and residuals are strictly uncorrelated. The latter may be thought of as a distributional IV-like objective but it generally does not estimate the causal model due to the fact that the anchor ${\varvec{A}}$ can also directly influence ${\varvec{H}}$ and $Y$ (see Fig. 1) and the conditional expectation function $\mathbb {E}[Y\vert {\varvec{X}}]$ is generally non-linear. It leads to some invariance of the score residuals across the values of the anchors ${\varvec{A}}$, and such an invariance property has been referred to as “diluted causality” (Bühlmann 2020).

The rest of this paper is organized as follows. In Sect. 2, we present the necessary theoretical background for linear $L_2$ anchor regression, transformation models and score residuals. Section 3 introduces distributional anchor regression and theoretical results on residual invariance and identifiability of the causal parameter in instrumental variable settings. Empirical results are presented in Sect. 4. We end with a discussion of our contribution in Sect. 5. In the appendix we present further details on notation, computation, score residuals and further empirical results on point-prediction performance of distributional anchor regression in comparison with linear $L_2$ and non-linear anchor boosting. We implement all methods and algorithms in the R language for statistical computing (R Core Team 2020) and the code is available on GitHub.

## Background

First, we introduce structural equation models (SEMs) before recapping linear anchor regression. In Sect. 2.3, we switch perspectives from modelling the conditional expectation to transformation models which capture the entire conditional distribution. The notation used in this work is described in Appendix A.

### Structural equation models

Let $Y$ be a response which takes values in $\mathbb {R}$, ${\varvec{X}}$ be a random vector of covariates taking values in $\mathbb {R}^p$, ${\varvec{H}}$ denotes hidden confounders with sample space $\mathbb {R}^d$, and ${\varvec{A}}$ are exogenous variables (called anchors, due to exogeneity; source node in the graph in Fig. 1) taking values in $\mathbb {R}^q$. The SEM governing linear anchor regression is given by

1 $$\begin{aligned} \begin{pmatrix} Y\\ {\varvec{X}}\\ {\varvec{H}}\end{pmatrix} \leftarrow {\mathbf {B}}\begin{pmatrix} Y\\ {\varvec{X}}\\ {\varvec{H}}\end{pmatrix} + {\mathbf {M}}{\varvec{A}}+ {\varvec{\varepsilon }}, \end{aligned}$$

with $(1+p+d) \times (1+p+d)$-matrix ${\mathbf {B}}$ which corresponds to the structure of the SEM in terms of a directed acyclic graph (DAG), the effect of ${\varvec{A}}$ enters linearly via the $(1 + p +d ) \times q$-matrix ${\mathbf {M}}$, and ${\varvec{\varepsilon }}$ denotes the error term with mutually independent components. The “$\leftarrow$” symbol is algebraically a distributional equality sign. It emphasizes the structural character of the SEM, saying that, *e.g.,* $Y$ is only a function of the parents of the node $Y$ in the structural DAG and the first entry in the additive component $({\mathbf {M}}{\mathbf {{ A}}}+ {\varvec{\varepsilon }})$.

The anchors ${\varvec{A}}$ may be continuous or discrete. In the special case of discrete anchors each level can be viewed as an “environment”.

We define perturbations as intervening on ${\varvec{A}}$, *e.g.,* by ${{\,\mathrm{do}\,}}({\varvec{A}}= {\varvec{a}})$, which replaces ${\varvec{A}}$ by ${\varvec{a}}$ in the SEM while leaving the underlying mechanism, *i.e.,* the coefficients in the SEM, unchanged. In this work we restrict ourselves to ${{\,\mathrm{do}\,}}$- (Pearl 2009) and ${{\,\mathrm{push}\,}}$-interventions (Markowetz et al. 2005) on ${\varvec{A}}$, which in turn lead to shifts in the distribution of ${\varvec{X}}$. In essence, ${{\,\mathrm{do}\,}}$-interventions replace a node in a graph with a deterministic value, whereas ${{\,\mathrm{push}\,}}$-interventions are stochastic and only “push” the distribution of the intervened random variable towards, *e.g.,* having a different mean. Since ${\varvec{A}}$ is exogenous and a source node in the graph, the specific type of intervention does not play a major role. Christiansen et al. (2021) show that under the above conditions OOD generalization is possible in linear models.

### Linear anchor regression

Linear $L_2$ anchor regression with its corresponding causal regularization estimates the linear regression parameter ${\varvec{\beta }}$ as

$$\begin{aligned} \hat{{\varvec{\beta }}} = \mathop {\mathrm {arg\,min}}\limits _{{\varvec{\beta }}} \bigg \{ \left\Vert ({{\,\mathrm{Id}\,}}- \varvec{\Pi }_{{\mathbf {A}}})({\varvec{y}}-{\mathbf {X}}{\varvec{\beta }})\right\Vert _2^2/n \ \\ +\,\gamma \left\Vert \varvec{\Pi }_{{\mathbf {A}}}({\varvec{y}}- {\mathbf {X}}{\varvec{\beta }})\right\Vert _2^2/n \bigg \}, \end{aligned}$$

where $0 \le \gamma \le \infty$ is a regularization parameter and $\varvec{\Pi }_{{\mathbf {A}}}= {\mathbf {A}}({\mathbf {A}}^\top {\mathbf {A}})^{-1}{\mathbf {A}}^\top$ denotes the orthogonal projection onto the column space of the anchors (Rothenhäusler et al. 2021). For $\gamma = 1$ one obtains ordinary least squares, $\gamma \rightarrow \infty$ corresponds to two-stage least squares as in instrumental variables regression and $\gamma = 0$ is partialling out the anchor variables ${\varvec{A}}$ (which is equivalent to ordinary least squares when regressing $Y$ on ${\varvec{X}}$ and ${\varvec{A}}$). Causal regularization encourages, for large values of $\gamma$, uncorrelatedness of the anchors ${\varvec{A}}$ and the residuals. As a procedure, causal regularization does not depend at all on the SEM in Eq. (1). However, as described below, the method inherits a distributional robustness property, whose formulation depends on the SEM in Eq. (1).

Rothenhäusler et al. (2021) establish the duality between the $L_2$ loss in linear anchor regression and optimizing a worst case risk over specific shift perturbations. The authors consider shift perturbations ${\varvec{\nu }}$, which are confined to be in the set

$$\begin{aligned} C_\gamma := \bigg \{{\varvec{\nu }}: {\varvec{\nu }}= {\mathbf {M}}{\varvec{\delta }}, \; {\varvec{\delta }} \text { independent } \text {of } {\varvec{\varepsilon }}, \;\\ \mathbb {E}\left[ {\varvec{\delta }}{\varvec{\delta }}^\top \right] \preceq \gamma \mathbb {E}\left[ {\mathbf {A}}{\mathbf {A}}^\top \right] \bigg \}, \end{aligned}$$

and which generate the perturbed response $Y^{\varvec{\nu }}$, and covariates ${\varvec{X}}^{\varvec{\nu }}$ via

$$\begin{aligned} \begin{pmatrix} Y^{\varvec{\nu }}\\ {\varvec{X}}^{\varvec{\nu }}\\ {\varvec{H}}^{\varvec{\nu }}\end{pmatrix} \leftarrow {\mathbf {B}}\begin{pmatrix} Y^{\varvec{\nu }}\\ {\varvec{X}}^{\varvec{\nu }}\\ {\varvec{H}}^{\varvec{\nu }}\end{pmatrix} + {\varvec{\nu }}+ {\varvec{\varepsilon }}. \end{aligned}$$

The set $C_\gamma$ contains all vectors which lie in the span of the columns of ${\mathbf {M}}$ and thus in the same direction as the exogenous contribution ${\mathbf {M}}{\varvec{A}}$ of the anchor variables. The average size and direction of the perturbations ${\varvec{\delta }}$ is limited by $\gamma$ and the centered anchors’ variance-covariance matrix. Now, the explicit duality between the worst case risk over all shift perturbations of limited size and the linear $L_2$ anchor loss is given by

2 $$\begin{aligned} \sup _{{\varvec{\nu }}\in C_\gamma }&\mathbb {E}\left[ (Y^{\varvec{\nu }}- ({\varvec{X}}^{\varvec{\nu }})^\top {\varvec{\beta }})^2\right] \nonumber \\&\quad =\mathbb {E}\left[ (({{\,\mathrm{Id}\,}}- P_{\varvec{A}})(Y-{\varvec{X}}^\top {\varvec{\beta }}))^2\right] \nonumber \\&\qquad +\gamma \mathbb {E}\left[ (P_{\varvec{A}}(Y- {\varvec{X}}^\top {\varvec{\beta }}))^2\right] , \end{aligned}$$

where $P_{\varvec{A}}= \mathbb {E}[\cdot \vert {\varvec{A}}]$ is the population analogue of $\varvec{\Pi }_{{\mathbf {A}}}$. We note that the right-hand side is the population analogue of the objective function in anchor regression. Hence, causal regularization in anchor regression provides guarantees for optimizing worst-case risk across a class of shift perturbations. The details are provided in Rothenhäusler et al. (2021).

### Transformation models

We now switch perspective from models for the conditional mean to modelling conditional distributions. Specifically, we consider transformation models (Hothorn et al. 2014). TMs decompose the conditional distribution of $Y\vert {\varvec{x}}$ into a pre-defined distribution function $F_Z$, with log-concave density $f_Z$, and a (semi-) parametric transformation function $h(y\vert {\varvec{x}})$, which is monotone non-decreasing in $y$

$$\begin{aligned} F_{Y\vert {\varvec{x}}}(y\vert {\varvec{x}}) = F_Z(h(y\vert {\varvec{x}})). \end{aligned}$$

This way, the problem of estimating a conditional distribution simplifies to estimating (the parameters of) the transformation function $h= F_Z^{-1} \circ F_{Y\vert {\varvec{x}}}$ (since $F_Z$, called inverse link, is pre-specified and parameter-free). Depending on the complexity of $h$, very flexible conditional distributions can be modelled. Hothorn et al. (2018) give theoretical guarantees for the existence and uniqueness of the transformation function $h$ for absolute continuous, count and ordered discrete random variables. For the sake of generality, $h$ is parametrized in terms of a basis expansion in the argument $y$ which can be as simple as a linear function in $y$ or as complex as a spline to model a smooth function in $y$.

In this work, we assume the transformation function for a continuous response can be additively decomposed into a linear predictor in ${\varvec{x}}$ and a smooth function in $y$ which is modelled as a Bernstein polynomial of order *P* with parameters ${\varvec{\theta }}\in \mathbb {R}^{P+1}$ (Hothorn et al. 2018), such that $h(y \vert {\varvec{x}}) = {\varvec{b}}_{\text {Bs},P}(y)^\top {\varvec{\theta }}+ {\varvec{x}}^\top \beta$. Monotonicity of ${\varvec{b}}_{\text {Bs},P}(y)^\top {\varvec{\theta }}$ and thereby of $h(y\vert {\varvec{x}})$ can then be enforced via the *P* linear constraints $\theta _1 \le \theta _2 \le \dots \theta _{P+1}$. In case of an ordinal response taking values in $\{y_1, y_2, \dots , y_K\}$, the transformation function is a monotone increasing step function, $h(y_k \vert {\varvec{x}}) = \theta _k + {\varvec{x}}^\top {\varvec{\beta }}$, for $k = 1, \dots , K - 1$ and the additional constraint $\theta _K = + \infty$. We summarize a transformation model based on its inverse link function $F_Z$, basis ${\varvec{b}}$, which may include covariates, and parameters ${\varvec{\vartheta }}$, such that $F_{Y\vert {\varvec{x}}}(y\vert {\varvec{x}}) = F_Z\left( {\varvec{b}}(y,{\varvec{x}})^\top {\varvec{\vartheta }}\right)$. For instance, for a transformation model with continuous response and explanatory variables ${\varvec{x}}$ we thus use ${\varvec{b}}(y,{\varvec{x}}) = ({\varvec{b}}_{\text {Bs},P}(y)^\top , {\varvec{x}}^\top )^\top$ and ${\varvec{\vartheta }}= ({\varvec{\theta }}^\top , {\varvec{\beta }}^\top )^\top$, yielding $h(y\vert {\varvec{x}}) = {\varvec{b}}_{\text {Bs},P}(y)^\top {\varvec{\theta }}+ {\varvec{x}}^\top {\varvec{\beta }}$. For a TM with ordinal response we substitute the Bernstein basis with a dummy encoding of the response, which we denote by ${\tilde{{\varvec{y}}}}$ (*e.g.,* Kook et al. 2022). Also note that the unconditional case is covered by the above formulation as well, by omitting all explanatory variables from the TM’s basis.

**Fig. 2:**
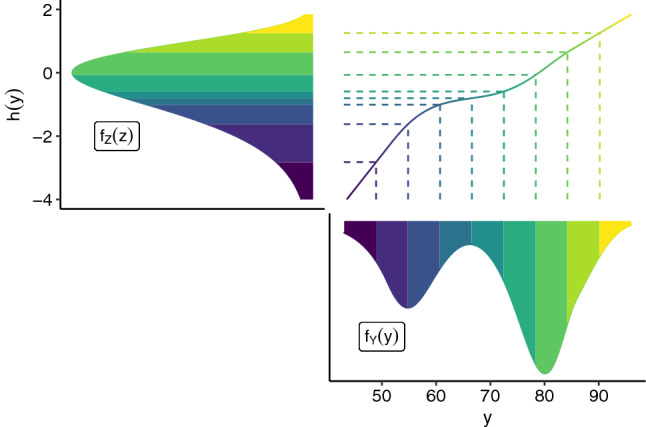
Illustration of an unconditional transformation model $(1 - \exp (-\exp (\cdot )), {\varvec{b}}_{\text {Bs},6}, {\varvec{\vartheta }})$ for the Old Faithful Geyser data (Azzalini and Bowman 1990) using a Bernstein polynomial basis expansion of order 6 for the transformation function, $h(y) = {\varvec{b}}_{\text{ Bs },6}(y)^\top \varvec{\vartheta }$. The colored regions indicate the transport of probability mass from $\mathbb {P}_Y$ (lower right) to $\mathbb {P}_Z$ (upper left) via the transformation function $h(y)$ (upper right). If $h$ is continuously differentiable, the density of $Y$ is given by $f_Y(y) = f_Z(h(y))h'(y)$

Figure 2 illustrates the intuition behind transformation models. The transformation function (upper right panel) transforms the complex, bimodal distribution of $Y$ (lower panel) to $F_Z= F_{{{\,\mathrm{MEV}\,}}}$, the standard minimum extreme value distribution (upper left panel). An analogous figure for ordinal outcomes is published in Kook et al. (2022, Fig. 1).

#### Definition 1

(*Transformation model, Definition 4 in* Hothorn et al. (2018)) The triple ($F_Z$, ${\varvec{b}}$, ${\varvec{\vartheta }}$) is called transformation model.

#### Example 1

(Linear regression) The normal linear regression model (Lm) is commonly formulated as $Y= \beta _0 + {\varvec{x}}^\top {\tilde{{\varvec{\beta }}}} + \varepsilon$, $\varepsilon \sim \mathcal {N}\left( 0, \sigma ^2\right)$, or $Y\vert {\varvec{x}}\sim {\mathcal {N}}\left( \beta _0 + {\varvec{x}}^\top {\tilde{{\varvec{\beta }}}}, \sigma ^2\right) .$ For a distributional treatment we write the above expression as

3 $$\begin{aligned} F_{Y\vert {\varvec{x}}}(y\vert {\varvec{x}}) = \varPhi \left( \frac{y- \beta _0 - {\varvec{x}}^\top {\tilde{{\varvec{\beta }}}}}{\sigma }\right) \end{aligned}$$

4 $$\begin{aligned} = {\varPhi }(\vartheta _1 + \vartheta _2 y- {\varvec{x}}^\top {\varvec{\beta }}), \end{aligned}$$

which can be understood as a transformation model by letting $\vartheta _1 = - \beta _0 / \sigma$, $\vartheta _2 = 1/\sigma$ and ${\varvec{\beta }}= {\tilde{{\varvec{\beta }}}} / \sigma$. Formally, it corresponds to the model

$$\begin{aligned} (F_Z, {\varvec{b}}, {\varvec{\vartheta }}) = \left( \varPhi , \left( 1, y, {\varvec{x}}^\top \right) ^\top , \left( \vartheta _1, \vartheta _2, -{\varvec{\beta }}^\top \right) ^\top \right) . \end{aligned}$$

Note that the baseline transformation, $h(y\vert {\varvec{X}}= 0)$, is constrained to be linear with constant slope $\vartheta _2$. Due to the linearity of $h$ and the choice $F_Z=\varPhi$, the modeled distribution of $Y\vert {\varvec{x}}$ will always be normal with constant variance. By parametrizing $h$ in a smooth way, we arrive at much more flexible conditional distributions for $Y\vert {\varvec{x}}$.

The parameters of a TM can be jointly estimated using maximum-likelihood. The likelihood can be written in terms of the inverse link function $F_Z$, which makes its evaluation computationally more convenient. For a single datum $(y, {\varvec{x}})$ with potentially censored response $y\in (\underline{y}, {\overline{y}}]$ the log-likelihood contribution is given by (Lindsey et al. 1996)

$$\begin{aligned}&\ell ({\varvec{\vartheta }}; y, {\varvec{x}}) = \\ {}&{\left\{ \begin{array}{ll} \log f_Z\left( {\varvec{b}}(y,{\varvec{x}})^\top {\varvec{\vartheta }}\right) + \log \left( {\varvec{b}}'(y,{\varvec{x}})^\top {\varvec{\vartheta }}\right) , &{}{} \text{ exact, } \\ \log F_Z\left( {\varvec{b}}\left( \overline{y},{\varvec{x}}\right) ^\top {\varvec{\vartheta }}\right) , &{}{} \text{ left, }\\ \log \left( 1 - F_Z\big ({\varvec{b}}(\underline{y},{\varvec{x}})^\top {\varvec{\vartheta }}\big ) \right) , &{}{} \text{ right, } \\ \log \left( F_Z\left( {\varvec{b}}\left( \overline{y},{\varvec{x}}\right) ^\top {\varvec{\vartheta }}\right) - F_Z\big ({\varvec{b}}(\underline{y},{\varvec{x}})^\top {\varvec{\vartheta }}\big ) \right) , &{}{} \text{ interval. } \end{array}\right. } \end{aligned}$$

The likelihood is always understood as conditional on ${\varvec{X}}$ when viewing the covariables as random. Allowing for censored observations is of practical importance, because in many applications the response of interest is not continuous or suffers from inaccuracies, which can be taken into account via uninformative censoring.

#### Example 2

(Lm, cont’d) For an exact datum $(y, {\varvec{x}})$ the log-likelihood in the normal linear regression model is given by

$$\begin{aligned} \ell (\vartheta _1, \vartheta _2, {\varvec{\beta }}; y, {\varvec{x}}) = \log \phi \big (\vartheta _1 + \vartheta _2 y- {\varvec{x}}^\top {\varvec{\beta }}\big ) + \log (\vartheta _2), \end{aligned}$$

using the density approximation to the likelihood (Lindsey et al. 1996). Here, $\phi$ denotes the standard normal density, and ${\varvec{b}}'(y,{\varvec{x}})^\top {\varvec{\vartheta }}= \frac{\partial {\varvec{b}}(y,{\varvec{x}})^\top {\varvec{\vartheta }}}{\partial y} = \vartheta _2$.

Now that we have established TMs and the log-likelihood function to estimate their parameters, we also need a more general notion of the residuals to formulate a causal regularizer for a distributional anchor loss. Most importantly, these residuals have to fulfill the same requirements as least squares residuals in the linear $L_2$ anchor loss. That is, they have to have zero expectation and a positive definite covariance matrix (Theorem 3 in Rothenhäusler et al. 2021). In the survival analysis literature, score residuals have received considerable attention, and fulfill the above requirements at least asymptotically (Lagakos 1981; Barlow and Prentice 1988; Therneau et al. 1990; Farrington 2000). We now define score residuals for the general class of transformation models.

#### Definition 2

(*Score residuals*) Let $(F_Z, {\varvec{b}}, {\hat{{\varvec{\vartheta }}}})$ be a TM with maximum-likelihood estimate ${\hat{{\varvec{\vartheta }}}}$. On the scale of the transformation function, add an additional intercept parameter $-\alpha$, to arrive at the TM

$$\begin{aligned} \left( F_Z, \left( {\varvec{b}}^\top , 1\right) ^\top , \left( {\hat{{\varvec{\vartheta }}}}^\top , -\alpha \right) ^\top \right) \end{aligned}$$

with distribution function

$$\begin{aligned} F_{Y\vert {\varvec{x}}}(y\vert {\varvec{x}}) = F_Z\left( {\varvec{b}}(y,{\varvec{x}})^\top {\hat{{\varvec{\vartheta }}}} - \alpha \right) . \end{aligned}$$

Because ${\hat{{\varvec{\vartheta }}}}$ maximizes the likelihood, $\alpha$ is constrained to 0. The score residual for a single datum $y \in (\underline{y}, {\bar{y}}]$ is now defined as

5 $$\begin{aligned} r := \frac{\partial }{\partial \alpha } \ell ({\varvec{\vartheta }}, \alpha ; y, {\varvec{x}}) \bigg |_{{\hat{{\varvec{\vartheta }}}}, \alpha \equiv 0}, \end{aligned}$$

which can be understood as the score contribution of a single observation to test $\alpha = 0$ for a covariate which is not included in the model. When viewed as a random variable, the vector of score residuals has mean zero asymptotically and its components are asymptotically uncorrelated (Farrington 2000).

The score residuals can be derived in closed form for a transformation model and observations under any form of uninformative censoring

6 $$\begin{aligned} r = {\left\{ \begin{array}{ll} - f_Z'\left( {\varvec{b}}(y,{\varvec{x}})^\top {\hat{{\varvec{\vartheta }}}}\right) \big /f_Z\left( {\varvec{b}}(y,{\varvec{x}})^\top {\hat{{\varvec{\vartheta }}}}\right) , &{}\text{ exact, } \\ - f_Z\left( {\varvec{b}}\left( \overline{y},{\varvec{x}}\right) ^\top {\hat{{\varvec{\vartheta }}}}\right) \big / F_Z\left( {\varvec{b}}\left( \overline{y},{\varvec{x}}\right) ^\top {\hat{{\varvec{\vartheta }}}}\right) , &{}\text{ left, }\\ f_Z\left( {\varvec{b}}(\underline{y},{\varvec{x}})^\top {\hat{{\varvec{\vartheta }}}}\right) \big / \left( 1 - F_Z\left( {\varvec{b}}(\underline{y},{\varvec{x}})^\top {\hat{{\varvec{\vartheta }}}}\right) \right) , &{}{} \text{ right, } \\ \left( f_Z\left( {\varvec{b}}(\underline{y},{\varvec{x}})^\top {\hat{{\varvec{\vartheta }}}}\right) - f_Z\left( {\varvec{b}}(\overline{y},{\varvec{x}})^\top {\hat{{\varvec{\vartheta }}}}\right) \right) \big / &{}{} \text{ interval. } \\ \quad \left( F_Z\left( {\varvec{b}}(\overline{y},{\varvec{x}})^\top {\hat{{\varvec{\vartheta }}}}\right) - F_Z\left( {\varvec{b}} (\underline{y},{\varvec{x}})^\top {\hat{{\varvec{\vartheta }}}}\right) \right) \end{array}\right. } \end{aligned}$$

#### Example 3

(Lm, cont’d) By including the addtitional intercept parameter in the normal linear model in Eq. (3), the score residuals are given by

$$\begin{aligned}&\frac{\partial }{\partial \alpha } \ell (\vartheta _1, \vartheta _2, {\varvec{\beta }}, \alpha ; y, {\varvec{x}}) \bigg |_{{\hat{\vartheta }}_1, {\hat{\vartheta }}_2, {\hat{{\varvec{\beta }}}}, \alpha \equiv 0}\\ {}&\quad =\frac{\partial }{\partial \alpha } \left\{ \log \phi \left( \vartheta _1 + \vartheta _2 y- {\varvec{x}}^\top {\varvec{\beta }}-\alpha \right) + \log (\vartheta _2) \right\} \bigg |_{{\hat{\vartheta }}_1, {\hat{\vartheta }}_2, {\hat{{\varvec{\beta }}}}, \alpha \equiv 0} \\ {}&\quad = {\hat{\vartheta }}_1 + {\hat{\vartheta }}_2 y- {\varvec{x}}^\top {\hat{{\varvec{\beta }}}} = \frac{y - {\hat{\beta }}_0 - {\varvec{x}}^\top \hat{{\tilde{{\varvec{\beta }}}}}}{{\hat{\sigma }}}. \end{aligned}$$

In this simple case the score residuals are equivalent to scaled least-square residuals, which underlines the more general nature of score residuals. In Sect. 3.1 and Appendix C, we give further examples and intuition on score residuals in non-linear and non-Gaussian settings.

We are now ready to cast transformation models into the framework of SEMs. Here, it is natural to view the response *Y* as a deterministic function of the transformed random variable $Z\sim F_Z$, which is given by the inverse transformation function $h^{-1}$ in the following definition.

#### Definition 3

(*Structural equation transformation model*) Let the conditional distribution of $Y\vert {\varvec{X}}, {\varvec{H}}, {\varvec{A}}$ be given by the transformation model $F_{Y\vert {\varvec{X}}, {\varvec{H}}, {\varvec{A}}} = F_Z\circ h$. The structural equation for the response is a deterministic function of ${\varvec{X}}$, ${\varvec{H}}$, ${\varvec{A}}$ and the exogenous $Z$, which, by definition, is distributed according to $F_Z$ and independent of $({\varvec{X}}, {\varvec{H}}, {\varvec{A}})$. Relationships other than the transformation function are assumed to be linear. Taken together, the following SEM defines a (partially) linear structural equation transformation model

$$\begin{aligned} Y&\leftarrow g(Z, {\varvec{X}}, {\varvec{H}}, {\varvec{A}}) := h^{-1}(Z\vert {\varvec{X}}, {\varvec{H}}, {\varvec{A}}) \\ {\varvec{X}}&\leftarrow {\mathbf {B}}_{{\varvec{X}}{\varvec{X}}} {\varvec{X}}+ {\mathbf {B}}_{{\varvec{X}}{\varvec{H}}} {\varvec{H}}+ {\mathbf {M}}_{\varvec{X}}{\varvec{A}}+ {\varvec{\varepsilon }}_{\varvec{X}}\\ {\varvec{H}}&\leftarrow {\mathbf {B}}_{{\varvec{H}}{\varvec{H}}} {\varvec{H}}+ {\mathbf {M}}_{\varvec{H}}{\varvec{A}}+ {\varvec{\varepsilon }}_{\varvec{H}}\\ {\varvec{A}}&\leftarrow {\varvec{\varepsilon }}_{\varvec{A}}\\ Z&\sim F_Z, \end{aligned}$$

where ${\varvec{\varepsilon }}_{\varvec{X}}, {\varvec{\varepsilon }}_{\varvec{H}}, {\varvec{A}}, Z$ are jointly independent.

As always, the structural equations are defined to hold as statements in distribution. By Corollary 1 in Hothorn et al. (2018), the transformation function $h$ and its inverse exist, are unique and monotone non-decreasing in $Y$ and $Z$, respectively. In contrast to the linear SEM in Eq. (1), the SEM in Definition 3 is set up involving a transformed response and a potentially non-linear inverse transformation *g*.

However, from the perspective of transformation models it is more natural to parametrize the transformation function $h$ instead of its inverse, because parameters in linear TMs are readily interpretable on this scale. For the empirical evaluation of the proposed estimator, we set up the transformation function as

7 $$\begin{aligned} h(Y\vert {\varvec{X}}, {\varvec{H}}, {\varvec{A}}) = {\varvec{b}}(y)^\top {\varvec{\theta }}- {\varvec{\beta }}^\top {\varvec{X}}- {\mathbf {B}}_{Y{\varvec{H}}}{\varvec{H}}- {\mathbf {M}}_{Y}{\varvec{A}}. \end{aligned}$$

**Fig. 3:**
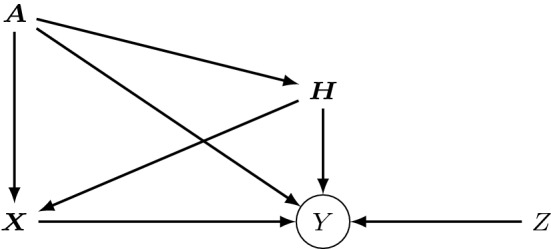
Structural equation model for a transformation model. Instead of setting up the SEM on the scale of $Y$, it is defined on the scale of the inverse transformation function $h^{-1}$. The conditional distribution of $Y:= h^{-1}(Z \vert {\varvec{X}}, {\varvec{H}}, {\varvec{A}})$ is still fully determined by $h$ and $F_Z$. The circle around $Y$ emphasizes that its distribution is a deterministic function of its parents

A graphical representation of the SEM in Definition 3 is shown in Fig. 3. The basis expansion ${\varvec{b}}(y)^\top {\varvec{\theta }}$ in Eq. (7) can be viewed as an intercept function, which fixes the overall shape of the transformation function. The remaining additive components of the transformation function, in turn, solely shift the transformation up- or downwards with the covariates. This may seem restrictive at first, however, all covariates influence not only the conditional mean, but all higher conditional moments of $F_{Y\vert {\varvec{X}}, {\varvec{H}}, {\varvec{A}}}$. We do not display the possibility that some components of ${\varvec{X}}$ directly influence each other, and likewise for ${\varvec{H}}$. In fact, in the simulations in Sect. 4, the coefficients ${\mathbf {B}}_{{\varvec{X}}{\varvec{X}}} = {\mathbf {B}}_{{\varvec{H}}{\varvec{H}}} = 0$.

Next, we will present our main proposal on distributional anchor regression to achieve robust TMs with respect to perturbations on the anchor variables ${\varvec{A}}$.

## Distributional anchor regression

We now formulate distributional anchor regression, for which we consider a distributional loss function, *i.e.,* the negative log-likelihood, which can take into account censored observations and captures the conditional distribution of $Y\vert {\varvec{X}}$, and a causal regularizer involving score residuals. We first give some intuition how to arrive at the distributional anchor loss, starting from the linear $L_2$ anchor loss. One can decompose the linear $L_2$ anchor loss

$$\begin{aligned}&L_2({\varvec{\beta }}; {\varvec{y}}, {\mathbf {X}}, {\mathbf {A}}) = \left\| ({{\,\mathrm {Id}\,}}- \varvec{\Pi }_{{\mathbf {A}}})({\varvec{y}}- {\mathbf {X}}{\varvec{\beta }})\right\| _2^2\\ {}&\quad + \gamma \left\| \varvec{\Pi }_{{\mathbf {A}}}({\varvec{y}}- {\mathbf {X}}{\varvec{\beta }})\right\| _2^2 \end{aligned}$$

into a squared error and a pure penalty term. We rewrite

$$\begin{aligned} L_2({\varvec{\beta }}; {\varvec{y}}, {\mathbf {X}}, {\mathbf {A}}) = \left\Vert {\varvec{y}}- {\mathbf {X}}{\varvec{\beta }}\right\Vert _2^2 + (\gamma - 1) \left\Vert \varvec{\Pi }_{{\mathbf {A}}}({\varvec{y}}- {\mathbf {X}}{\varvec{\beta }})\right\Vert _2^2, \end{aligned}$$

which is a sum of the squared-error loss and a causal regularizer involving the $L_2$ norm of the residuals $({\varvec{y}}- {\mathbf {X}}{\varvec{\beta }})$ projected linearly onto the space spanned by the columns of ${\mathbf {A}}$ to encourage uncorrelatedness between the residuals and the anchor variables. The cross-terms when expanding the $L_2$ norm vanish because $\varvec{\Pi }_{{\mathbf {A}}}$ is an orthogonal projection. Now an intuitive choice for the distributional anchor regression loss would be

$$\begin{aligned} L({\varvec{\beta }}; {\varvec{y}}, {\mathbf {X}}, {\mathbf {A}}) \propto - \sum _{i=1}^n \ell ({\varvec{\beta }}; y_i, {\varvec{x}}_i) + (\gamma - 1) \left\Vert \varvec{\Pi }_{{\mathbf {A}}}{\varvec{r}}\right\Vert _2^2, \end{aligned}$$

where the negative log-likelihood induced by a transformation model, $\ell (\cdot )$, replaces the squared error loss and, most importantly, ${\varvec{r}}$ denotes the vector of score residuals as defined in Sect. 2.3. Thus, the loss encourages uncorrelatedness between the anchor variables and the score residuals, particularly for large values of $\gamma$. The origin and importance of score residuals is outlined in Appendix B. We now give a definition for the distributional anchor loss.

### Definition 4

(*Distributional anchor loss*) Consider a linear TM and its SEM, as in Definition 3. Then, the empirical distributional anchor loss is defined as

$$\begin{aligned} L({\varvec{\vartheta }}; {\varvec{y}}, {\mathbf {X}}, {\mathbf {A}}, \xi ) = - \sum _{i=1}^{n} \ell ({\varvec{\vartheta }}; y_i, {\varvec{x}}_i)/n + \xi \left\Vert \varvec{\Pi }_{{\mathbf {A}}}{\varvec{r}}\right\Vert _2^2/n, \end{aligned}$$

where $\ell (\cdot )$ denotes the log-likelihood induced by a TM, ${\varvec{r}}$ denotes the vector of score residuals and $\xi \in [0, +\infty )$ controls the extent of causal regularization. As mentioned earlier, the log-likelihood is conditional on ${\varvec{X}}$.

### Example 4

*(Lm, cont’d)* For normal linear regression with constant variance, the linear $L_2$ anchor loss and the distributional anchor loss are equivalent. This is because

$$\begin{aligned}&L(\vartheta _1, \vartheta _2, {\varvec{\beta }}; {\varvec{y}}, {\mathbf {X}}, \xi ) \\&\quad = - \sum _{i=1}^n \left\{ \log \phi (\vartheta _1 + \vartheta _2 y_i - {\varvec{x}}_i^\top {\varvec{\beta }}) \right\} / n - \log (\vartheta _2) \\&\qquad + \xi \Vert \varvec{\Pi }_{{\mathbf {A}}}(\vartheta _1 + \vartheta _2{\varvec{y}}- {\mathbf {X}}{\varvec{\beta }})\Vert _2^2/n \\&\quad = \Vert {\varvec{y}}- \beta _0 - {\mathbf {X}}{\tilde{{\varvec{\beta }}}}\Vert _2^2 / (2 \sigma ^2 n) \\&\qquad + \xi \Vert \varvec{\Pi }_{{\mathbf {A}}}({\varvec{y}}- \beta _0 - {\mathbf {X}}{\tilde{{\varvec{\beta }}}})\Vert _2^2 / (\sigma ^2 n) + C. \end{aligned}$$

Absorbing all additive constants into *C* and factoring out the variance renders the above objective equivalent to the linear $L_2$ anchor loss for $\xi = (\gamma - 1) / 2$.

Relaxing the assumptions of linear $L_2$ anchor regression towards censored outcomes and non-linear expectation functions comes at the cost of some theoretical guarantees. In the following, we discuss which aspects of the residual distribution remain invariant in distributional anchor regression and issues in identification of the causal parameter when the anchors are valid instruments.

### Residual invariance

When considering the case $\xi \rightarrow \infty$, the solution to the distributional anchor loss in the population version is a parameter ${\varvec{\vartheta }}^{\rightarrow \infty } = \lim _{\xi \rightarrow \infty } {\varvec{\vartheta }}(\xi )$, which fulfills ${{\,\mathrm{Corr}\,}}({\varvec{A}}, {\varvec{r}}({\varvec{\vartheta }}^{\rightarrow \infty })) = 0$ (assuming $\mathbb {E}[{\varvec{A}}] = 0$). As in Bühlmann (2020), we consider the set

8 $$\begin{aligned} I = \{{\varvec{\vartheta }}\mid \mathbb {E}[{\varvec{A}}r({\varvec{\vartheta }})] = 0 \}, \end{aligned}$$

which contains solutions with zero correlation between anchors and score residuals. In particular, ${\varvec{\vartheta }}^{\rightarrow \infty } \in I$. We then arrive at a result similar to Proposition 5.1 in Bühlmann (2020).

#### Proposition 1

Consider an anchor TM as in Definition 3 and assume $\mathbb {E}[{\varvec{A}}{\varvec{A}}^\top ]$ to be positive definite. Assume that $\mathbb {E}[r({\varvec{\vartheta }}) \vert {\varvec{A}}] = \mu _{\varvec{\vartheta }}+ {\varvec{\zeta }}_{\varvec{\vartheta }}^\top {\varvec{A}}$ is linear in ${\varvec{A}}$, where $r(\cdot )$ denotes the score residual, $\mu _{\varvec{\vartheta }}\in \mathbb {R}$, and ${\varvec{\zeta }}_{\varvec{\vartheta }}\in \mathbb {R}^{q}$. Then, for any ${\varvec{\vartheta }}\in I$ we have for every ${{\,\mathrm{do}\,}}$-intervention on ${\varvec{A}}$ with ${{\,\mathrm{do}\,}}({\varvec{A}}= {\varvec{a}})$

9 $$\begin{aligned} \mathbb {E}[r({\varvec{\vartheta }}) \vert {{\,\mathrm{do}\,}}({\varvec{A}}= {\varvec{a}})] \equiv \mu _{\varvec{\vartheta }}, \end{aligned}$$

which is independent of the (deterministic or random) value ${\varvec{a}}$.

Note that the linearity assumption in the conditional expectation function of the residuals holds if the anchors are discrete (Bühlmann 2020, Corollary 5.1). The proof of Proposition 1 is analogous to the one given in Bühlmann (2020, Prop. 5.1). Proposition 1 shows that, similar to non-linear anchor regression, the first moment of the score residuals conditional on the anchors is invariant. However, it does not state any invariance properties for higher moments or the entire residual distribution, which can be achieved in linear $L_2$ anchor regression (Rothenhäusler et al. 2021, Theorem 3).

To build further intuition about residual invariance in distributional anchor regression, the following example shows that score residuals are equivalent to martingale residuals in a parametric version of the Cox proportional hazard model (Barlow and Prentice 1988; Therneau et al. 1990). Further examples of score residuals for transformation models with probit and logit link are given in Appendix C.

#### Example 5

(Cox regression) The Cox proportional hazards (PH) model (Cox 1975) is defined via

$$\begin{aligned} \lambda (y\vert {\varvec{x}}) = \lambda _0(y)\exp ({\varvec{x}}^\top {\varvec{\beta }}), \end{aligned}$$

where $\lambda$ denotes the hazard function which is assumed to consist of a baseline hazard $\lambda _0$ and a multiplicative contribution of the covariates. The Cox PH model can be understood as a transformation model when choosing $F_Z(z) = 1 - \exp (-\exp (z))$. Then we have

$$\begin{aligned} h(y\vert {\varvec{x}}) = \log \Lambda (y\vert {\varvec{x}}) = \log \Lambda _0(y) + {\varvec{x}}^\top {\varvec{\beta }}, \end{aligned}$$

where $\Lambda (y\vert {\varvec{x}}) = \int _0^y\lambda (u\vert {\varvec{x}})\mathrm {d}u$ denotes the cumulative hazard and $\Lambda _0$ the cumulative baseline hazard function. For a potentially right censored observation $(y, {\varvec{x}}, \delta )$, where $\delta \in \{0, 1\}$ denotes the event indicator (with 1 specifying an exact and 0 a right censored observation), the likelihood contribution is given by

$$\begin{aligned} \ell ({\varvec{\vartheta }},{\varvec{\beta }}; y, {\varvec{x}}, \delta ) = \delta \left[ h(y\vert {\varvec{x}}) + \log h'(y\vert {\varvec{x}})\right] - \exp (h(y\vert {\varvec{x}})). \end{aligned}$$

The score residual can be derived by including and restricting the additional intercept $\alpha \equiv 0$ in the model in (5) or via the general form given in (6)

$$\begin{aligned} r = \delta - \exp ({\hat{h}}(y\vert {\varvec{x}})) = \delta - {\hat{\Lambda }}(y\vert {\varvec{x}}), \; r \in (-\infty , 1]. \end{aligned}$$

The above expression for the score residual is equivalent to martingale residuals, which quantify the discrepancy between the event indicator (“observed”) and the estimated cumulative hazard (“predicted”), somewhat analogous to least squares residuals. In contrast to least square residuals, martingale residuals have a skewed distribution (see, *e.g.,* Aalen et al. 2008).

For distributional anchor regression, de-correlating the martingale residuals from the anchors (*e.g.,* different countries or hospitals) stabilizes out-of-distribution prediction in the sense that the first moment of the martingale residuals is invariant across intervention values of the anchors, due to Proposition 1.

**Table 1:** Transformation models used to illustrate distributional anchor regression

Name	Transformation model	Constraints	Type response
Lm	$\left( \varPhi , \left( 1, y, {\varvec{x}}^\top \right) ^\top , \left( \theta _1, \theta _2, -{\varvec{\beta }}^\top \right) ^\top \right)$	$\theta _2 > 0$	Continuous
c-probit	$\left( \varPhi , \left( {\varvec{b}}_{\text{ Bs },P}^\top , {\varvec{x}}^\top \right) ^\top , \left( {\varvec{\theta }}^\top , -{\varvec{\beta }}^\top \right) ^\top \right)$	$\theta _1 \le \dots \le \theta _{P+1}$	Continuous
c-logit	$\left( F_{{{\,\mathrm {SL}\,}}}, \left( {\varvec{b}}_{\text{ Bs },P}^\top , {\varvec{x}}^\top \right) ^\top , \left( {\varvec{\theta }}^\top , -{\varvec{\beta }}^\top \right) ^\top \right)$	$\theta _1 \le \dots \le \theta _{P+1}$	Continuous
o-logit	$\left( F_{{{\,\mathrm {SL}\,}}}, \left( {\tilde{{\varvec{y}}}}^\top , {\varvec{x}}^\top \right) ^\top , \left( {\varvec{\theta }}^\top , -{\varvec{\beta }}^\top \right) ^\top \right)$	$\theta _1< \dots< \theta _{K - 1} < \theta _K = +\infty$	Ordinal

$F_{{{\,\mathrm{SL}\,}}}= {{\,\mathrm{expit}\,}}$ denotes the standard logistic distribution. By ${\tilde{{\varvec{y}}}}$ we denote the dummy encoded response, ${\tilde{{\varvec{y}}}} = {\varvec{e}}(k)$, for $Y$ taking class $y_k$, $k = 1, \ldots , K$. Here, ${\varvec{e}}(k)$ denotes the *k*th unit vector. In the experiments, the basis functions for $y$ are Bernstein polynomials with maximum order *P*, ${\varvec{b}}_{\text {Bs},P}(y) \in \mathbb {R}^{P+1}$. Because the transformation function $h(y) = {\varvec{b}}(y)^\top {\varvec{\vartheta }}$ must be monotone non-decreasing, we require some constraints on the parameters of the transformation function

### Identifiability of the causal parameter

Linear $L_2$ anchor regression is equivalent to instrumental variable regression for $\gamma \rightarrow \infty$ (see Sect. 2). As a consequence, the causal parameter is identified as long as the anchors are valid instruments and the conditional expectation $\mathbb {E}[Y\vert {\varvec{X}}]$ is linear (Angrist et al. 1996). However, as soon as one leaves the linear regime, restrictive moment conditions have to be employed, namely $\mathbb {E}[Y - f({\varvec{X}}) \vert {\varvec{A}}] = 0$. Then, *f* is commonly fitted using a generalized method of moments estimator (see, *e.g.,* Foster (1997) for the logistic linear case).

The causal parameter is not identified in anchor TMs. The score-residual condition ${\varvec{\vartheta }}^{\rightarrow \infty } \in I$ is not directly related to a moment condition as described above. In fact, score residuals can be interpreted as the slope of the negative log-likelihood contribution of a single observation evaluated at the maximum-likelihood estimate (MLE). Thus, they also take into account the curvature of the log-likelihood (*i.e.,* the variance of the prediction instead of first moments only). An empirical evaluation of the non-identifability is given in Sect. 4.2.

We note that neither (non-) linear $L_2$ nor distributional anchor regression is targeted for estimating the causal parameter. Instead, these methods aim to stabilize and improve worst-case prediction error under perturbations. Proposition 1 leads to an interpretation of stability: ${\varvec{\vartheta }}^{\rightarrow \infty }$ is the parameter for which the first conditional moment of the residuals given the anchors remains invariant.

In the following Section, we will empirically evaluate the prediction performance of transformation models estimated under the distributional anchor loss. We empirically investigate non-identification of the causal parameter in two simulation scenarios in Sect. 4.2. Computational details for fitting TMs using the distributional anchor loss, are given in Appendix D.

## Empirical results

We begin the section by describing the experimental setup in the application and simulation studies and then present the results in Sects. 4.1 and 4.2. We consider median housing prices in the BostonHousing2 dataset (Harrison and Rubinfeld 1978) to illustrate an application of anchor TMs in normal linear regression (Lm), which assumes normality and equal variances. To lift these assumptions, a continuous outcome probit (c-probit) regression is used to model more complex, skewed distributions, which are typical for housing prices. Then, we use a continuous outcome logistic (c-logit exact) model, which enables more easily interpretable shift effects on the log-odds scale. Lastly, we show the c-logit (censored) model which now takes into account the censored observations in the BostonHousing2 dataset and retains interpretability of the parameters on the log-odds scale. Furthermore, the proposed distributional framework for anchor regression is evaluated in simulation studies for Lm, c-probit and ordered logistic regression (o-logit). A summary of the models used to empirically evaluate anchor TMs is given in Table 1.

**Fig. 4:**
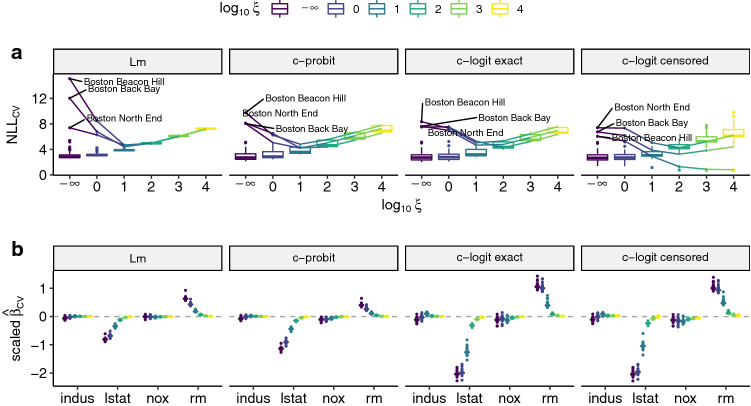
Leave-one-environment-out cross validation under increasing causal regularization for the BostonHousing2 data, with town as anchors. A linear (Lm), continuous probit (c-probit) and continuous logit (c-logit, using the exact and censored response) model is fitted on 91 towns and used to predict the left out town. **a** Mean out-of-sample NLL for the left-out census tracts. Beacon Hill, Back Bay and North End are consistently hardest to predict. Consequently, for these towns the cross-validated NLL improves with increasing causal regularization up to a certain point. For the majority of the remaining towns prediction performance decreases. We thus indeed improve worst-case prediction, in analogy to Eq. (2). Note that $\log _{10} \xi = - \infty$ corresponds to the unpenalized model. **b** Scaled regression coefficients, which are interpretable as difference in means (Lm), difference in transformed means (c-probit) and log odds-ratios (c-logit) per standard deviation increase in a covariate. Solely the c-logit (censored) model accounts for right-censored observations. With increasing causal regularization the estimates shrink towards zero, indicating that town may be a weak instrument (see Appendix E)

To evaluate prediction performance, we study the average negative log-likelihood (NLL). The NLL is a strictly proper scoring rule and thus encourages honest distributional forecasts (Good 1952; Gneiting and Raftery 2007). In addition, the NLL is comparable across nested TMs and different choices for $F_Z$.

To study “worst-case” prediction performance, the quantile function of the individual NLL contributions is studied. Here, larger quantiles reflect harder-to-predict observations, since the model assigns a low likelihood at the estimated parameters conditional on the observation. This approach is the distributional analog to studying quantiles of the squared errors (Rothenhäusler et al. 2021) or the absolute deviations (Bühlmann 2020) in (non-) linear $L_2$ anchor regression.

### Application: BostonHousing2

For the BostonHousing2 data we wish to predict corrected median housing prices (cmedv) from several socio-economical and environmental factors ($n = 506$). These include per capita crime rates (crim), average number of rooms (rm), and nitric oxide concentration (nox) among others. Each observation corresponds to a single census tract in Boston. Individual cities will serve as anchors in this example because they are plausibly exogenous factors that induce heterogeneity in the observed covariates and housing prices.

“Leave-one-environment-out” (LOEO) cross validation is used to demonstrate the change in estimated regression coefficients and NLL comparing a plain model without causal regularization ($\xi = 0$) to three different anchor TMs over a large range of causal regularization (Fig. 4). For some of the left-out towns the conditional distribution of cmedv will differ from the training distribution and contain unseen perturbations. In this case, housing prices of this town will be harder to predict, leading to a worse cross-validated NLL compared to the environments which are not perturbed. We hypothesize, an anchor TM should improve prediction performance for the census tracts in these hard-to-predict towns, in analogy to the distributional robustness results implied by Eq. (2), whereas it should perform worse than a plain TM for environments with only mild perturbations.

First, a linear model assuming homoscedasticity and conditional normality is used to estimate the conditional distribution of cmedv depending on the socio-economic factors described above. A notable reduction in the observed worst-case loss is already observed for mild causal regularization ($\xi \in \{1, 10\}$) without losing too much predictive performance for the other environments (Fig. 4a). For stronger causal regularization, the mean cross-validated NLL becomes gradually worse. However, assuming a symmetric distribution for prices ignores the typical skewness of these outcomes. The c-probit model estimates a non-linear basis expansion in the response and thus relaxes the homoscedasticity and conditional normality assumption. When switching from $F_Z= \varPhi$ to $F_Z= {{\,\mathrm{expit}\,}}$, the interpretation of $h$ changes from a latent normal to a logistic scale. The former has no direct interpretation, whereas for the latter, ${\varvec{\beta }}$ can be interpreted as log odds-ratios. A similar gain in terms of worst-case CV NLL is observed for the c-probit model compared to Lm.

**Fig. 5:**
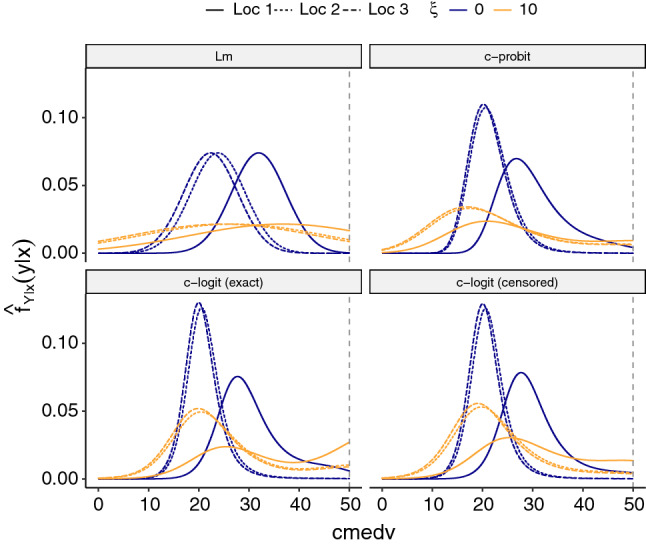
Density estimates for the three census tracts (Loc 1, Loc 2, Loc 3) in Boston Beacon Hill, the hardest to predict town in terms of LOEO cross-validated NLL for $\xi = 10$ (*cf.* Fig. 4). The dashed gray line indicates the observed outcomes for all three locations, which were all censored at $\$50'000$. Lm assumes equal variances and conditional normality, whereas c-probit loosens this assumption leading to more accurate, skewed distributions. Only c-logit (censored) takes into account right censoring in the data and puts a smaller probability density on $\$50'000$ than the c-logit (exact) model, which ignores the censoring

Figure 5 shows the predicted conditional densities for the three observations in Boston Beacon Hill and emphasizes the importance of modelling cmedv using a right-skewed distribution. The densities are shown for the regularized transformation model ($\xi = 10$) and the unregularized model ($\xi = 0$). For all regularized models, a flatter (*i.e.,* more uncertain) distribution is predicted, putting more probability mass on the values beyond $\$50'000$.

A disadvantage of switching from a linear probit to a non-linear probit model is the loss of interpretability of the individual regression coefficients (*e.g.,* Fahrmeir et al. 2007).

However, also this disadvantage can be overcome in the framework of anchor TMs by specifying a different inverse link function, $F_Z$, while keeping the basis expansion in the outcome equally flexible. The c-logit (exact) model allows the interpretation of ${\hat{{\varvec{\beta }}}}$ on the log-odds scale and shows a similar gain in worst-case prediction to c-probit. However, the housing prices above $\$50'000$ (cmedv = 50) are right-censored in the BostonHousing2 data, which is commonly ignored in analyses, but crucial to capture the uncertainty in predicting the skewed outcome (Gilley and Kelley Pace 1996). The c-logit (censored) model now takes into account right censoring of the observations and still yields regression coefficients that are interpretable as log odds-ratios (Lohse et al. 2017). Indeed, for census tract Loc 1 the censored c-logit model reflects the higher uncertainty implied by the censored responses compared to the c-probit or the exact c-logit model (Fig. 5). Note how c-logit (exact) attributes high density to cmedv $=50$ by mistakenly treating the censored observations as exact, because the likelihood contribution is the density instead of the area under the density to the right of the censored observation.

Taking into account right censoring apparently facilitated out-of-sample prediction for Boston Beacon Hill, Back Bay and North End, but the improvement through causal regularization diminished slightly compared to c-probit or Lm (Fig. 4a).

Scaled coefficient estimates for all three models are shown in Fig. 4b. With increasing amount of causal regularization, all estimates shrink towards 0, which indicates town may be a weak instrument (Imbens and Rosenbaum 2005), for more details see Appendix E. However, intermediate amounts of causal regularization yield estimates for which anchors and score residuals are somewhat de-correlated and still lead to the desired robustness against perturbations in unseen environments.

### Simulations

In this section, the results of the simulation scenarios are presented. The scenarios along with parameters for the SEMs used to simulate from the models in Table 1 are summarized in Table 2.

#### Experimental setup

We begin with a comparison of linear $L_2$ anchor regression and the distributional version of linear anchor regression in scenario la, which was first used to study anchor regression in Bühlmann (2020). The non-linear scenario nla also stems from Bühlmann (2020), which we use to show how shift transformation models can estimate non-linear conditional expectation function, albeit for their linear model formulation in the covariates. For the last two scenarios iv1 and iv2, the IV assumptions hold, *i.e.,* the anchors influence only ${\varvec{X}}$. Scenario iv1 showcases discrete anchors and a continuous response and a non-linear transformation function, which we model by a continuous probit regression. Scenario iv2 features an ordinal response and a more detailed simulation, including various shift strengths. In scenarios la, iv1 and iv2, the effect from ${\varvec{X}}$ to $Y$ is denoted by ${\varvec{\beta }}$, whereas the non-linear *f* is used in scenario nla. For the data generating processes that involve transformation models, the transformation function $h$ is specified. For ordinal responses the number of classes, *K*, and for continuous outcomes, the maximum order of the Bernstein basis, *P*, determines the number of parameters for the baseline transformation. The parameters of the Bernstein basis are fixed by applying the transformation function $h$ to a $(P+1)$-vector of evenly spaced values in the desired support of $Y$. In turn, such a basis approximation leads to a distribution approximation for the true distribution of $Y$ which improves as *P* increases. However, the transformation function is constrained to be monotone non-decreasing, which makes a parsimonious parametrization sufficient.

**Table 2:**
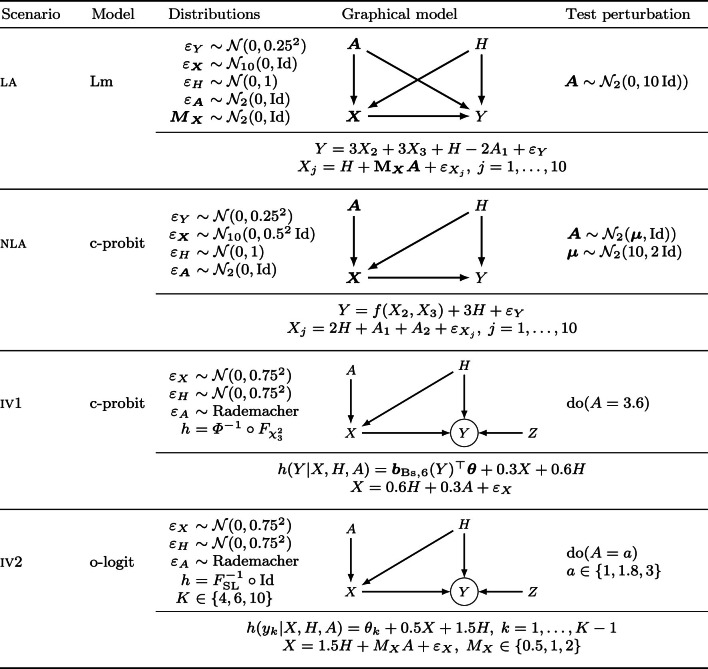
Simulation scenarios used to empirically evaluate distributional anchor regression

Scenarios la and nla are adapted from Bühlmann (2020) and will be used to evaluate linear and continuous probit anchor TMs. The eight covariates omitted in the table in both scenarios are noise covariates, *i.e.,* $\beta _j = 0, \; j \ne 2,3$. In nla, $f({\varvec{X}}) = X_2 + X_3 + \mathbbm {1}(X_2 \le 0) + \mathbbm {1}(X_2 \le -0.5)\mathbbm {1}(X_3 \le 1)$. Both use $n_{\text {train}} = 300$ and $n_{\text {test}} = 2000$ observations. In the iv scenarios the instrumental variables assumptions hold, because the anchor neither influences the hidden confounders nor the response. The scenarios generalize Example 2 in Rothenhäusler et al. (2021) to anchor TMs with a continuous outcome (iv1) and an ordinal outcome (iv2). Both use $n_{\text {train}} = 1000$ and $n_{\text {test}} = 2000$ observations. A schematic DAG is shown together with the equations for all endogenous variables underneath. Note that for scenario nla which was taken from Bühlmann (2020), the data are not generated from the c-probit model but via the structural equation given below the DAG. For scenario iv1, in contrast, the data are generated via the c-probit model using the order 6 Bernstein polynomial basis (*i.e.,* the best approximation to $h$ is used)

#### Scenario la

**Fig. 6:**
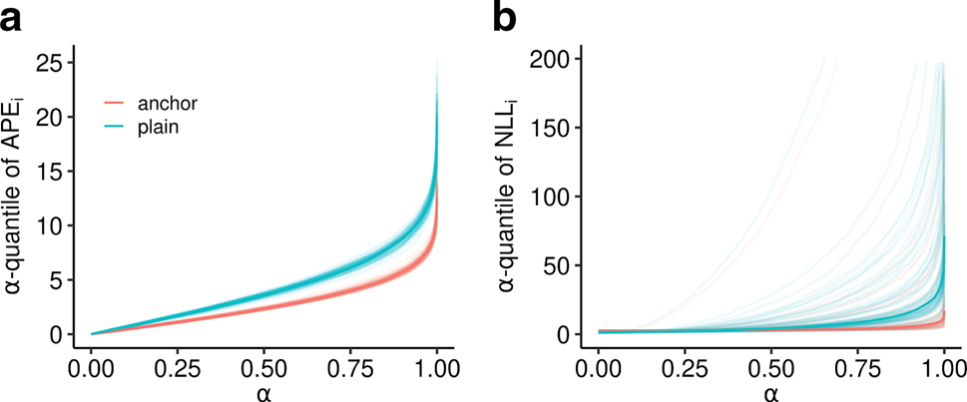
Test performance (thin lines) over 100 simulations for scenario la with $n_{\text {train}} = 300$ and $n_{\text {test}} = 2000$. Median test performance over all simulations is indicated by the thick line. The $\alpha$-quantiles of test absolute prediction error $\text {APE} := |y- {\hat{y}} |$, where $\hat{y}$ denotes the conditional median, is shown for linear $L_2$ anchor regression (**a**) using $\gamma = 13$ and the negative log-likelihood contributions for distributional (conditionally Gaussian) linear anchor regression (**b**) with $\xi = (\gamma - 1) / 2 = 6$. The two models are equivalent up to estimating the residual variance via maximum likelihood in the distributional anchor TM. The change in perspective from an $L_2$ to a distributional loss requires different evaluation metrics, of which the log-likelihood, being a proper scoring rule, is the most natural choice

The linear anchor scenario la was first presented in Bühlmann (2020) for the linear $L_2$ anchor loss. The performance gain of using anchor regression compared to a plain linear model is shown in Fig. 6 for the linear $L_2$ anchor loss (**a**) and the distributional anchor loss (**b**).

A performance gain across all quantiles of the log-likelihood contributions can be observed. However, the larger the quantile, the higher the performance gain. The extent of causal regularization was chosen based on the theoretical insight that, in a multivariate normal model, $\gamma$ can be interpreted as the quantile of a $\chi ^2_1$ distribution, which relates the expected size of the unobserved perturbations to the conditional mean squared error given the anchors (Lemma 1 in Rothenhäusler et al. 2021). The variability in the NLL’s quantile function in Fig. 6b appears to be larger than for the absolute prediction error (in panel a). We attribute this to the sensitivity of the NLL towards worst-case prediction errors, *i.e.,* for likelihood contributions close to zero, the NLL quickly diverges to minus infinity. The APE as a measure of central tendency is not as sensitive to worst-case prediction errors.

#### Scenario nla

**Fig. 7:**
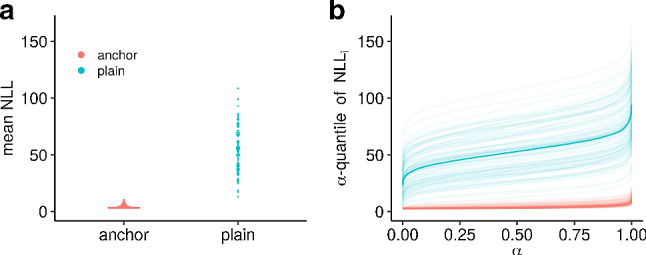
Test performance over 100 simulations for scenario nla with $n_{\text {train}} = 300$ and $n_{\text {test}} = 2000$. Mean (**a**) and $\alpha$-quantiles of the negative log-likelihood contributions (**b**) for the c-probit anchor TM. The test data are generated under strong push-interventions on the distribution of the anchors (*cf.* Table 2). The strength of causal regularization was chosen as $\xi = 6$

In scenario nla, which features non-linear anchor regression, a continuous probit model is fitted. Figure 7 shows a vast gain in performance over all quantiles of the NLL, comparable to what was observed in Bühlmann (2020) with $L_2$ anchor boosting for quantiles of the absolute prediction error.

This gain in performance can be explained in the causal generalization framework of Christiansen et al. (2021), because the causal function linearly extends outside the support of ${\varvec{X}}_{\text {train}}$. Note that although the graphical model representation suggests that the assumptions of IV regression hold, the conditional expectation is non-linear. Additional simulations for a mis-specified normal linear anchor TM are given in Appendix F, to warrant the use of a non-linear model.

In some applications, a point prediction may be more desirable than a distributional forecast. Anchor TMs can produce point estimates via the conditional quantile function, *e.g.,* the conditional median. However, in these settings we recommend (non-) linear $L_2$ anchor regression which is tailored specifically towards conditional mean estimation. In Appendix F, we present additional results for scenario nla to compare the performance of these point estimates from anchor TMs with results from $L_2$ anchor boosting (Bühlmann 2020) using a combined linear model, random forest base learner for reference. In essence, the conditional median predictions from anchor TM show a similar gain in worst-case absolute prediction error and can compete with the conditional mean predictions obtained from $L_2$ anchor boosting in this scenario.

#### Scenario iv1

In scenario la the anchors influence the response, violating the instrumental variable assumptions. Scenario iv1 explores binary anchors as valid instruments, while the baseline transformation ${\varvec{b}}_{\text {Bs},6}(y)^\top {\varvec{\theta }}$ is non-linear.

Note that although the model formulation is linear in ${\varvec{\beta }}$, the conditional expectation function may be non-linear, because of the non-linear transformation function. This scenario is inspired by Example 2 in Rothenhäusler et al. (2021) and translates it into a transformation model SEM from Definition 3 for continuous but non-normal outcomes.

**Fig. 8:**
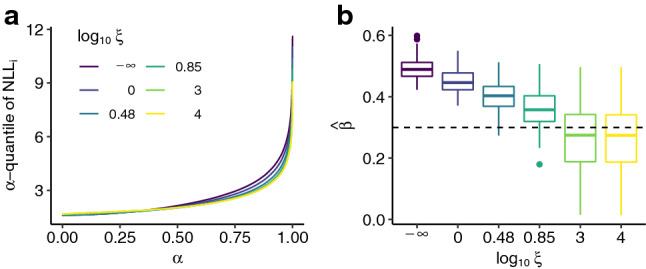
Test performance over 100 simulations for scenario iv1 with $n_{\text {train}} = 1000$ and $n_{\text {test}}=2000$. Quantiles of the individual negative log-likelihood contributions (**a**) and estimates of $\beta$ (**b**) for increasingly strong causal regularization. The ground truth is indicated by a dashed line. The test data are generated under the intervention ${{\,\mathrm{do}\,}}({\varvec{A}}= 3.6)$

The test data were generated using ${{\,\mathrm{do}\,}}(A=3.6)$, for which a better predictive performance under stronger causal regularization was observed (Fig. 8a). Additionally, although ${\varvec{A}}$ is a valid instrument, the causal parameter is biased for larger $\xi$ (Fig. 8b), due to the non-linear conditional expectation $\mathbb {E}[Y\vert X]$. Additional simulations for a mis-specified normal linear anchor TM fitted to data generated under scenario iv1 are given in Appendix F.

#### Scenario iv2

The instrumental variable assumptions hold also in the last scenario iv2. However, the response’s distribution is now ordered categorical and more varying parameters are considered, including the number of classes of the response, the strength of the instruments and the perturbations in the test data (*cf.* Table 2). Note that the ordinal response may be viewed as an interval censored version of an underlying latent continuous response with common censoring points. This point of view is especially useful for sampling from such a model and understanding it as a transformation model.

**Fig. 9:**
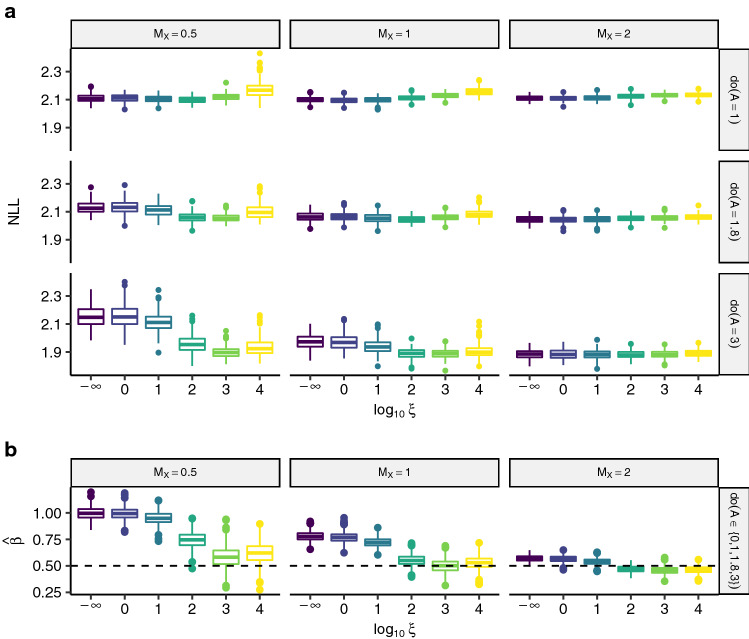
Test performance and coefficient estimates over 200 simulations for scenario iv2. Because the results are comparable for differing sample sizes and numbers of classes, solely the results for $n_{\text {train}} = 1000$ and $K = 10$ are displayed. **a**: Test log-likelihood contributions for varyingly strong instruments (columns) and perturbation sizes (rows). **b**: Parameter estimates ${\hat{\beta }}$ for all intervention scenarios together, since they do not influence estimation. The simulated ground truth $\beta = 0.5$ is indicated with a dashed line

Figure 9 depicts test NLL alongside the estimated shift parameter, ${\hat{\beta }}$. Also here, in case of strong perturbations anchor regression protects against unseen perturbations for larger $\xi$ (*e.g.,* ${{\,\mathrm{do}\,}}(A = 1.8)$ and ${{\,\mathrm{do}\,}}(A = 3)$ for $\mathrm {M}_{X} = 0.5$) resulting in improved test predictions. However, if the shift perturbations are not innovative, test prediction suffers with increasing amounts of causal regularization (*e.g.,* ${{\,\mathrm{do}\,}}(A = 1)$ for $\mathrm {M}_{X} = 2$). Note the interplay between the strength of the anchors, $\mathrm {M}_X$, and the strength of the shift interventions. For larger $\mathrm {M}_{X}$, the training data becomes more heterogeneous and the larger shifts are not as innovative, resulting in a weaker performance of anchor TMs for increasing $\xi$. Again, the estimated shift parameter is biased (Fig. 9b).

The additional simulation results for $K = 4$ and $K = 6$ are shown in Appendix F. The conclusions are unaffected at those values for *K*. However, having a bounded sample space for the response can be problematic, namely if shift perturbations are extremely large and the model is linear in ${\varvec{A}}$. Then, it may happen that the response’s marginal distribution is extremely skewed towards class 1 or *K*. Most prominently, this problem appears in the binary case.

## Discussion and outlook

The proposed method of distributional anchor regression generalizes (non-) linear anchor regression beyond the assumptions of normality and homoscedasticity and beyond estimating solely a conditional mean.

In an exemplary analysis of the BostonHousing2 data we have illustrated the flexibility of anchor TMs and demonstrated a gain in prediction performance in terms of worst-case cross validated log-likelihood, while preserving interpretability and appropriately accounting for censored observations. The simulations show comparable results to established linear and non-linear anchor regression models under both IV and invalid IV scenarios and extend the notion of invariance between residuals and environments to other than continuous responses. Although anchor TMs are generally unable to recover the causal parameter, we argue that the “diluted causal” (Bühlmann 2020) parameter, ${\hat{{\varvec{\beta }}}}^{\rightarrow \infty } := {\hat{{\varvec{\beta }}}}(\xi )$ as $\xi \rightarrow \infty$, is interesting in its own right, for it induces invariance between anchors and the first conditional moment of the score residuals. In this sense, it allows robust test predictions in the presence of distributional shifts. Much like the causal parameter, the diluted causal parameter leads to (aspects of) invariant conditional distributions across environments. Even though the powerful causal interpretation is lost, distributional anchor regression yields models that allow causally flavored interpretations in terms of stabilization and robustification across environments.

Anchor TMs estimate the whole conditional distribution and thus enable robust prediction of a multitude of responses, which we demonstrated for (censored) continuous and ordered categorical responses. Possible extensions of anchor TMs are numerous. For instance, other types of responses include count and time-to-event data. The framework of anchor TMs contains a fully parametric version of the Cox proportional hazard model (Hothorn et al. 2018), although an extension to classical survival models is also possible. For instance, the Cox proportional hazard model (Cox 1972) can be fitted by substituting the likelihood for the partial likelihood (Cox 1975) in the distributional anchor loss, while the score residuals are equivalent to martingale residuals (*cf.* Appendix B; Barlow and Prentice 1988; Therneau et al. 1990). As in high-dimensional linear and non-linear anchor regression, anchor TMs could be fitted under a lasso penalty (Tibshirani 1996). The idea of using a different class of residuals can also be translated to other model classes, such as deviance residuals for GLMs, as long as the theoretical requirements discussed in Sect. 3 are met.

In terms of future work, further theoretical investigation of the distributional anchor loss, such as bounds on the generalization error, is warranted. So far we restricted distributional regression to linear (in ${\varvec{x}}$) TMs because of their already highly flexible nature and simplicity in the considered DGPs. However, more complex experimental designs require, for instance, random effects or time-varying effects of covariates in time-to-event data. Taken together, anchor TMs lay the foundation for future work on distributional extensions of anchor regression.

## Figures and Tables

**Fig. 10:**
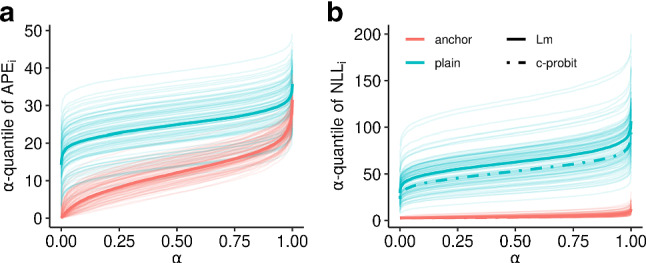
Test performance (quantile functions) over 100 simulations for scenario nla with the same parameters as in Fig. 7, but using a mis-specified linear $L_2$ anchor (**a**) and normal linear anchor transformation (**b**) model. The quantile function of the absolute prediction errors (**a**) and negative log-likelihood (**b**) contributions are shown with $\gamma = 13$ and $\xi = 6$, respectively. The point-wise median over all simulations is indicated by a thick line. As a reference, we show the point-wise median NLL of the c-probit model from Fig. 7 in **b**

**Fig. 11:**
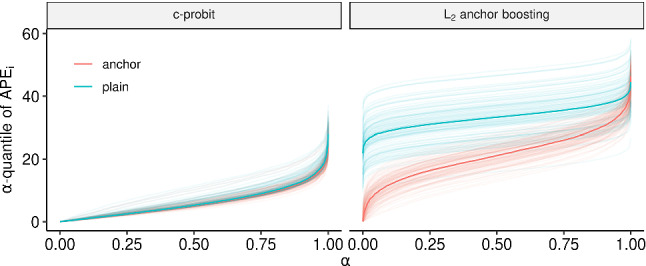
Test performance (quantile functions) over 100 simulations for scenario nla with the same parameters as in Fig. 7, using conditional mean predictions from $L_2$ anchor boosting ($\gamma = 13$) and conditional median predictions from a c-probit model ($\xi = 6$). The point-wise median over all simulations is indicated by a thick line

**Fig. 12:**
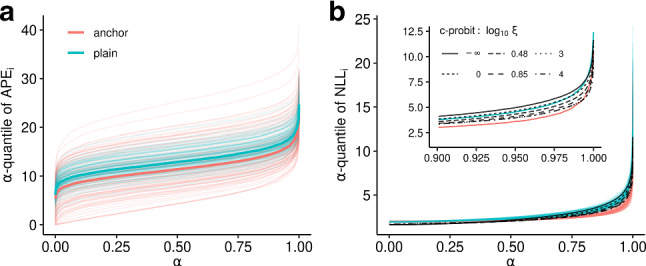
Test performance (quantile functions) over 100 simulations for scenario iv1 with the same parameters as in Fig. 8, but using a mis-specified linear $L_2$ anchor (**a**) and normal linear anchor transformation (**b**) model. The quantile function of the absolute prediction errors (**a**) and negative log-likelihood (**b**) contributions are shown with $\gamma = 15$ and $\xi = 7$, respectively. The point-wise median over all simulations is indicated by a thick line. The original point-wise median NLL of the correctly specified c-probit model from Fig. 8 is depicted in black

**Fig. 13:**
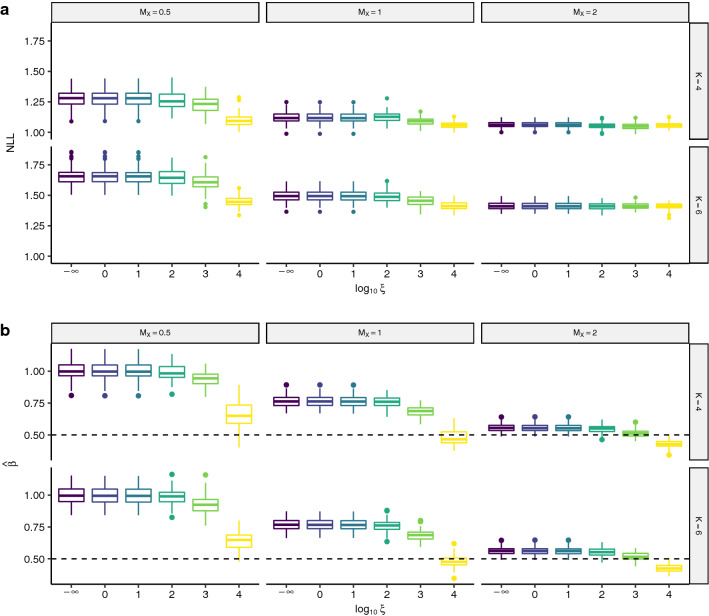
Simulation results for scenario iv2 for $K \in \{4, 6\}$ repeated 100 times. **a** The average test NLL is displayed for each simulation, varying $M_X$ and constant ${{\,\mathrm{do}\,}}(A = 3)$. **b** Coefficient estimates for each model, where the simulated ground truth ($\beta = 0.5$) is indicated by a dashed line. For details, see Fig. 9

## A Notation

Random variables are written in uppercase italic, $Y$, and realizations thereof in lowercase, $y$. When stacked to a vector of *n* observations, we write ${\varvec{y}}= (y_1, \dots , y_n)^\top$. Random vectors are written like random variables, but in bold, ${\varvec{X}}$, and realizations thereof in lowercase bold, ${\varvec{x}}$. Stacked to a matrix for *n* observations, we write ${\mathbf {X}}= ({\varvec{x}}_1^\top , \dots , {\varvec{x}}_n^\top )^\top \in \mathbb {R}^{n\times p}$. Matrices are written in bold, uppercase, ${\mathbf {A}}$, vectors in bold italic lowercase, ${\varvec{a}}$. The probability measure of a random variable $Y$ is denoted by $\mathbb {P}_Y$. Coefficient matrices in the SEMs are denoted by ${\mathbf {M}}$ for the anchors ${\varvec{A}}$ and by ${\mathbf {B}}$ for all other components. When restricting the coefficient matrix ${\mathbf {B}}$ to a single component, *e.g.,* the effect of ${\varvec{X}}$ on $Y$, we write ${\mathbf {B}}_{Y{\varvec{X}}}$. Because for ${\mathbf {M}}$ it is clear from context, we omit the ${\varvec{A}}$ in the index.

## B Background on score residuals

Stigler (2016) considers residuals the seventh “pillar of statistical wisdom”, which highlights their conceptual importance. Here, we will briefly justify the use of the score residual in transformation models based on the work of Lagakos (1981), who introduced martingale residuals for survival analysis under interval censoring. Farrington (2000) presents a summary of the different types of residuals used in survival analysis. The scope of score residuals in transformation models is to generalize the notion of a residual to a wider class of models, namely TMs, and allow for all kinds of uninformative censoring and response types.

In Definition 2, we note that the score residual can be interpreted as the score contribution of a newly introduced intercept parameter, which is constrained to zero. This is equivalent to formulating a score test for $\alpha = 0$ for a covariate that is not yet included in the model. Since $\alpha$ is constrained to zero in the whole procedure, we do not need to evaluate the model under the alternative hypothesis. Note also, that we restrict $\alpha$ to an intercept term on the scale of the transformation function. Farrington gives a compelling argument why one should do so. This connection is drawn next.

As an adjustment of Cox-Snell residuals (Cox and Snell 1968), Lagakos proposes a centered version of Cox-Snell residuals. Barlow and Prentice (1988) later drew the connection to stochastic calculus and coined the term “martingale residual” (see also Therneau et al. 1990). For interval and right censored, as well as exact responses, Lagakos residuals have a direct connection to score statistics (Farrington 2000). The setting is based in survival analysis, but the connection to transformation models will become apparent. Lagakos starts from a general proportional hazards model where

$$\begin{aligned} \lambda (t \vert {\varvec{x}}) = \lambda (t) \exp \left( {\varvec{x}}^\top {\varvec{\beta }}\right) \end{aligned}$$

denotes the hazard function depending on time *t* and covariates ${\varvec{x}}$, which can be decomposed into a product of a baseline hazard function $\lambda (t)$ and the exponential function of a linear predictor in the covariates ${\varvec{\beta }}$. The cumulative hazard function is then defined as

$$\begin{aligned} \Lambda (t \vert {\varvec{x}}) = \int _0^t \lambda (u \vert {\varvec{x}}) du. \end{aligned}$$

From there, the cdf can be recovered as

$$\begin{aligned} F_T(t \vert {\varvec{x}}) = 1 - \exp \left( -\Lambda (t)\exp \left( {\varvec{x}}^\top {\varvec{\beta }}\right) \right) . \end{aligned}$$

By definition $\Lambda (t) > 0 \; \forall t$, hence

$$\begin{aligned} F_T(t \vert {\varvec{x}}) = 1 - \exp \left( -\exp \left( \log \Lambda (t) + {\varvec{x}}^\top {\varvec{\beta }}\right) \right) , \end{aligned}$$

which is a transformation model with Minimum-Extreme-Value inverse-link, $F_Z = F_{{{\,\mathrm{MEV}\,}}}$, and the transformation function, $h(t) = \log \Lambda (t)$, is the log cumulative baseline hazard. Farrington now assumes that the baseline hazard function belongs to a family ${\mathcal {F}}$ that is closed under scaling by a factor $\gamma > 0$, *i.e.,* 

$$\begin{aligned} \Lambda (t |x) \in {\mathcal {F}}\Rightarrow \gamma \Lambda (t \vert {\varvec{x}}) \in {\mathcal {F}}. \end{aligned}$$

The Lagakos residual is now defined as

$$\begin{aligned} \hat{r}^L = \frac{\partial }{\partial \alpha } \ell \bigg |_{{\hat{{\varvec{\beta }}}},\hat{S}_0} \end{aligned}$$

for $\alpha = \log \gamma$ and $\hat{S}_0$ being the estimated survivor curve under ${\varvec{x}}= 0$, *i.e.,* $\hat{S}_0 = \exp (-\hat{\Lambda }(t \vert {\varvec{x}}= 0))$. This translates to the family of log (cumulative) hazard functions being closed under addition of $\alpha = \log \gamma \in \mathbb {R}$, which is exactly the case for transformation models. This step corresponds to adding and constraining a new intercept term to zero on the scale of the transformation function. Lagakos residuals behave like the usual score statistic, *i.e.,* they have mean zero asymptotically and are asymptotically uncorrelated.

## C Score residual forms

In Sect. 2, we have seen the equivalence between score and least square residuals for the normal linear regression model. In Sect. 3, we have shown that martingale residuals are score residuals for exact and right censored observations in a Cox PH transformation model. In the following, we present more examples of commonly appearing score residuals.

### Example 6

(Normal distribution with non-linear transformation function) When choosing $F_Z= \varPhi$ and a non-linear transformation function, *e.g.,* 

10 $$\begin{aligned} h(y\vert {\varvec{x}}) = h_0(y) - {\varvec{x}}^\top {\varvec{\beta }}, \end{aligned}$$

the score residual of an exact response is

$$\begin{aligned} r = {\hat{h}}_0(y) - {\varvec{x}}^\top {\hat{{\varvec{\beta }}}}. \end{aligned}$$

Thus, we arrive at a least-squares residual for the transformed response. Here, $r \in (-\infty , \infty )$.

### Example 7

(Logistic distribution with non-linear transformation function) Choosing $F_Z= {{\,\mathrm{expit}\,}}$ and the same transformation function as in (10), the score residual of an exact response is

$$\begin{aligned} r = 2 {{\,\mathrm {expit}\,}}\left( {\hat{h}}_0(y) - {\varvec{x}}^\top {\hat{{\varvec{\beta }}}}\right) - 1. \end{aligned}$$

Thus, in case the model predicts $y$ well (*i.e.,* ${\hat{h}}_0(y) - {\varvec{x}}^\top {\hat{{\varvec{\beta }}}}$ is close to zero, analogously to Example 6), the residual will be close to zero too. Note that here $r \in [-1, 1]$. For a right censored observation $y\in (\underline{y}, \infty )$, the score residual is simply the estimated probability

$$\begin{aligned} r = {{\,\mathrm {expit}\,}}\left( {\hat{h}}_0(\underline{y}) - {\varvec{x}}^\top {\hat{{\varvec{\beta }}}}\right) = \mathbb {P}\left( Y\le \underline{y} \big \vert {\varvec{x}}; {\hat{h}}_0, {\hat{{\varvec{\beta }}}}\right) , \end{aligned}$$

and for a left censored observation $y\in (-\infty , {\bar{y}}]$

$$\begin{aligned} r&= - \left( 1 - {{\,\mathrm {expit}\,}}\left( {\hat{h}}_0({\overline{y}}) - {\varvec{x}}^\top {\hat{{\varvec{\beta }}}}\right) \right) \\&= -\mathbb {P}\left( Y> {\overline{y}} \big \vert {\varvec{x}}; {\hat{h}}_0, {\hat{{\varvec{\beta }}}}\right) . \end{aligned}$$

Again, a good prediction should put little probability mass on either of the above two events and thus yield a small score residual. For the right censored case the residual is restricted to $r \in [0, 1]$, whereas for the left censored case to $r \in [-1, 0]$, which confirms the intuition that one can err only in one direction for such responses.

In the examples above, stabilizing the residuals across values of the anchors in distributional anchor regression can be expected to improve worst-case prediction under perturbations of the anchors.

## D Computational details

Anchor TMs, simulation scenarios and visualizations are written in the R language for statistical computing (Version 3.6.3, R Core Team 2020). To implement distributional anchor regression, note that the transformation model log-likelihood is concave w.r.t. ${\varvec{\vartheta }}$, unless all observations are right censored. The penalty resulting from the quadratic ${\varvec{r}}^\top \varvec{\Pi }_{{\mathbf {A}}}{\varvec{r}}$ is convex if ${\varvec{r}}$, from Definition 2, is affine or convex (Boyd and Vandenberghe 2004). In case of exact observations in Lm and c-probit, the resulting penalty is convex and thus solvable by a constrained convex optimizer. Kook and Hothorn (2021) implement regularized transformation models under elastic net penalties in **tramnet**, using the domain-specific language optimizer **CVXR** (Fu et al. 2020). From **tramnet**, we use the TM likelihood implementation. For the interval censored or right censored models (o-logit, c-logit) we fit the models using (stochastic) gradient descent (SGD) from package **TensorFlow** (Abadi et al. 2015). The implementation of the interval censored log-likelihood for ordinal TMs was taken from Kook et al. (2022) and we used SGD with the Adam optimizer (Kingma and Ba 2015) with learning rate $10^{-3}$, batch size 250 and 200 epochs. Parameters were initialized with the maximum likelihood estimate for $\xi = 0$ obtained via tram::Polr() (Hothorn 2020).

## E Invalid instruments and shrinkage of estimates

In the linear IV setting an instrument is called weak if it has little impact on the explanatory variables ${\varvec{X}}$. Consequently, a two-stage least squares procedure will yield homogeneous predictions for ${\varvec{X}}$ in the first stage, because

$$\begin{aligned} {\varvec{X}}\perp {\varvec{A}}\implies \varvec{\Pi }_{{\mathbf {A}}}{\mathbf {X}}\equiv {\text{ const. }} \end{aligned}$$

This leads to regressing the response on a matrix with constant columns, making it impossible to separate ${\varvec{\beta }}$ from an intercept. Thus, in case of weak instruments, the resulting estimates ${\hat{{\varvec{\beta }}}}$ will shrink towards 0 as $\xi \rightarrow \infty$ when using a causal regularizer. This may explain the effect seen for the BostonHousing2 data in Sect. 4.1 and makes the diluted causal parameter impossible to study, because it is equal to constant 0. However, intermediary amounts of causal regularization yield a benefit in terms of worst-case LOEO CV (*cf.* Fig. 4).

## F Additional simulation results

In this appendix, we report additional simulation results to more clearly substantiate the empirical findings in the main text.

### F.1 Scenario NLA

To show that the DGP for scenario nla is sufficiently challenging to warrant a non-linear anchor regression model, we fit a mis-specified linear $L_2$ anchor regression with $\gamma = 13$ and normal linear anchor TM with $\xi = 6$ to the data. As mentioned in the main text, the non-linear conditional expectation function $f({\varvec{x}}) = \mathbb {E}[Y\vert {\varvec{X}}={\varvec{x}}]$ extends linearly outside the training support and thus linear anchor regression performs only slightly worse (Fig. 10). In absolute terms, the mis-specified plain Lm performs worse than the c-probit model (*cf.* the dot-dashed line in Fig. 10b), whereas the anchor regression models perform similar.

Point estimates for anchor TMs can be obtained via the conditional quantile function, *e.g.,* the conditional median. However, we strongly recommend using models tailored towards conditional mean estimation in anchor regression, such as $L_2$ anchor boosting (Bühlmann 2020). In Fig. 11, the performance of anchor TMs (c-probit, $\xi = 6$) in terms of point prediction is compared with anchor boosting ($m_{\text {stop}} = 50$, $\gamma = 13$). Obtaining a conditional mean prediction in anchor TMs is cumbersome, because it involves numeric integration over incomplete densities.

The conditional median seems to perform reasonably well, even compared to conditional mean prediction in anchor boosting (assuming symmetric errors, the two prediction errors can be directly compared). However, the benefit of using distributional anchor regression is more strongly reflected in the NLL and not the APE, since the first is a proper scoring rule evaluating a probabilistic instead of a point prediction.

The performance gain in terms of NLL for c-probit remains the same as in Fig. 7. We refrain from comparing the anchor TM NLL against an anchor boosting NLL for which one could assume a Gaussian likelihood, because the method is not intended for distributional predictions.

### F.2 Scenario iv1

In a similar vein, to show that the DGP for scenario iv1 is sufficiently challenging, we again fit a mis-specified linear $L_2$ anchor regression with $\gamma = 13$ and normal linear anchor TM with $\xi = 6$ to the data. There is a performance gain in favor of linear anchor regression over the plain linear model for both APE and NLL. However, for small quantiles (up until the median), the mis-specified model performs worse than the c-probit model (*cf.* Fig. 8). The mis-specified model nevertheless exerts robustness properties and shows better performance than the correctly specified model for quantiles larger than 0.5 (but excluding the maximum). The DGP in scenario iv1 can be deemed sufficiently challenging to show the benefits of non-linear, distributional extensions of linear $L_2$ anchor regression. The mis-specified models still improve worst-case prediction, but suffer from worse performance for those observations that are “easy” to predict for the non-linear model (Fig. 12).

### F.3 Scenario iv2

Here, we show additional simulation results for scenario iv2 using a different number of classes for the ordinal outcome, namely $K = 4$ and $K = 6$. We restrict ourselves to the perturbation ${{\,\mathrm{do}\,}}(A = 3)$ (Fig. 13). The same main conclusions as for $K = 10$ can be drawn. However, responses with bounded sample space suffer from a common problem in linear model formulations if the shift perturbations are overly large. In this case, the response’s marginal distribution becomes extremely skewed towards class 1 or *K*.
